# Internet search results correlate with seasonal variation of sarcoidosis

**DOI:** 10.1186/s12890-021-01602-7

**Published:** 2021-07-13

**Authors:** Amanda Stanton, Steven J. Katz

**Affiliations:** Department of Medicine, University of Alberta, Edmonton, AB Canada

**Keywords:** Sarcoidosis, Google, Season, Variation, Internet

## Abstract

**Background:**

The etiology and pathophysiology of sarcoidosis remains unclear, with epidemiologic studies limited by its relatively low prevalence. The internet has prompted patients to seek information about medical diagnoses online; Google Trends provides access to an anonymized version of this data, which has a new role in epidemiology. We hypothesize that there is seasonal variation in the relative search interest of sarcoidosis, which would suggest seasonal variation in the incidence of sarcoidosis.

**Methods:**

Google Trends was used to assess the relative search volume from 2010 to 2020 for “sarcoidosis” and “sarcoid” in 7 countries. ANOVA with multiple comparisons was performed to compare the mean relative search volume by month and by season for each country, with a *p*-value less than 0.05 indicating statistical significance.

**Results:**

Our analysis revealed a significant seasonal variation in search popularity in 4 of the 7 countries and in the Northern Hemispheric countries combined. Direct comparison showed search terms to be more popular in spring, specifically March & April, than in the winter. Southern Hemisphere data was not statistically significant but showed a trend towards a nadir in December and a peak in September and October.

**Conclusions:**

Overall, these findings suggest seasonal variation with a possible peak in spring and nadir in winter. This supports the hypothesis that sarcoidosis has seasonal variation and is more commonly diagnosed in spring, but more evidence is needed to support this, as well as investigation into the pathophysiology of sarcoidosis to explain this phenomenon.

**Supplementary Information:**

The online version contains supplementary material available at 10.1186/s12890-021-01602-7.

## Background

Sarcoidosis is an inflammatory condition of unknown etiology, characterized by non-caseating granulomas. More than 90% of patients will have pulmonary involvement, but sarcoidosis can manifest in almost any body system, including the lymph nodes, joints, skin, heart, and GI tract [1]. Most individuals present with symptoms before the age of 40, and it is slightly more common in females and non-smokers [2, 3]. Incidence is variable depending on ethnicity and geography, ranging from about 1/100,000 in Japan [4] and Southern Europe to 35.5/100,000 amongst the African-American population [1]. Genetic and environmental influences have been hypothesized as factors in the pathogenesis of sarcoidosis [1], and the condition has been anecdotally considered to be more common in the winter-spring months [5]. This has been supported by a study based on 345 cases of sarcoidosis in Minnesota over four decades which observed lowest incidence in autumn [6], as well as a smaller study in Taiwan of 56 individuals in which about two-thirds were diagnosed in the winter and early spring months [7]. However, the mechanism behind this phenomenon has yet to be determined.

With improving access to information through the Internet, patients commonly seek answers to their medical questions online [8]. Google Trends is a program which allows for analysis of a “largely unfiltered sample of actual [Google] search requests”, dating as far back as 2004 [9]. With trillions of Google searches every year, Trends is “one of the world’s largest real-time data sets” [10]. Data can be used to compare the relative search term volume between or within countries, within a time period, or compared to another search term. Any given data point has been “normalized to the time and location of a query,… scaled on a range of 0 to 100” [9]; as such, a value of 100 indicates the data point with the highest relative search volume, necessitating the remainder of the data points be lower than this. Therefore, this cannot be used to infer absolute search volumes.

The term “infodemiology” encompasses the use of information obtained online “in the analysis, detection, and forecasting of diseases and epidemics, and in predicting human behaviour” in health [11]. Infodemiologic studies are becoming increasingly more popular as a research tool in medicine; in particular, Google Trends data has been shown to correlate with prevalence of certain diseases, such as multiple sclerosis [12]. More evidence has surfaced showing Google Trends as a potentially useful tool for quickly elucidating epidemiologic patterns in infectious and non-infectious diseases, as well as a method of surveillance [12]. This study aims to examine search trends for sarcoidosis and determine if there is a seasonal pattern.

## Methods

The manuscript does not contain clinical studies or patient data. Google Trends data was extracted for each of the terms “Sarcoid” and “Sarcoidosis”, using filters for “Web Searches”, “All Categories”, “Worldwide” and from January 2010 to July 2020 (Please see Additional Files 1 and 2). The search volumes in the worldwide dataset were separated by country and analysed to determine which countries have an adequate search volume to use individual datasets for analysis. From this, the ten countries with the highest relative search volume were isolated; countries were selected for analysis if they satisfied this criteria for both “sarcoid” and “sarcoidosis”. Countries were excluded if their relative search volume was less than 35, meaning less than 35% of the value of the country with the highest relative search volume. Countries were also excluded if their population was less than 3 million, according to the United Nations 2019 Population Division Estimates [13]. Data was extracted for each of these countries individually, with the remaining 3 filters constant, allowing for relative search volume to be displayed monthly.

The resulting data for each search term, as well as a combined dataset of the two terms, were grouped by month and by season. March to May was designated as Spring; June to August as Summer, September to November as Fall, and December to February as Winter in the Northern Hemisphere. These would correlate to Fall, Winter, Spring, and Summer in the Southern Hemisphere, respectively. Data from the countries in the Northern Hemisphere and those in the Southern Hemisphere were combined to create another two groups. ANOVA with multiple comparisons was used to compare the mean relative search volume in each set between seasons and between months. A result with a p-value of less than 0.05 was considered statistically significant.

The data from Google Trends is anonymized and in the public domain [9]. For this reason, and that no patients were involved in this study, ethics approval by the University of Alberta Research Ethics Board was waived.

## Results

Seven countries were identified, which met the study’s inclusion criteria: Australia, New Zealand, South Africa, Canada, Ireland, the United Kingdom (UK), and the United States (USA). The relative search volume for “sarcoid” and “sarcoidosis” for these countries is found in Table 1. The “Southern Hemisphere” and “Northern Hemisphere” datasets were composed of the first three and remaining four countries, respectively.

**Table 1:** Relative search volumes of countries meeting study inclusion criteria

Country	Query interest (%, compared to most popular country)	
	Sarcoid	Sarcoidosis
Ireland	100	100
United Kingdom	95	56
New Zealand	75	56
Australia	72	56
United States	56	76
Canada	41	52
South Africa	35	40

**Fig. 1:**
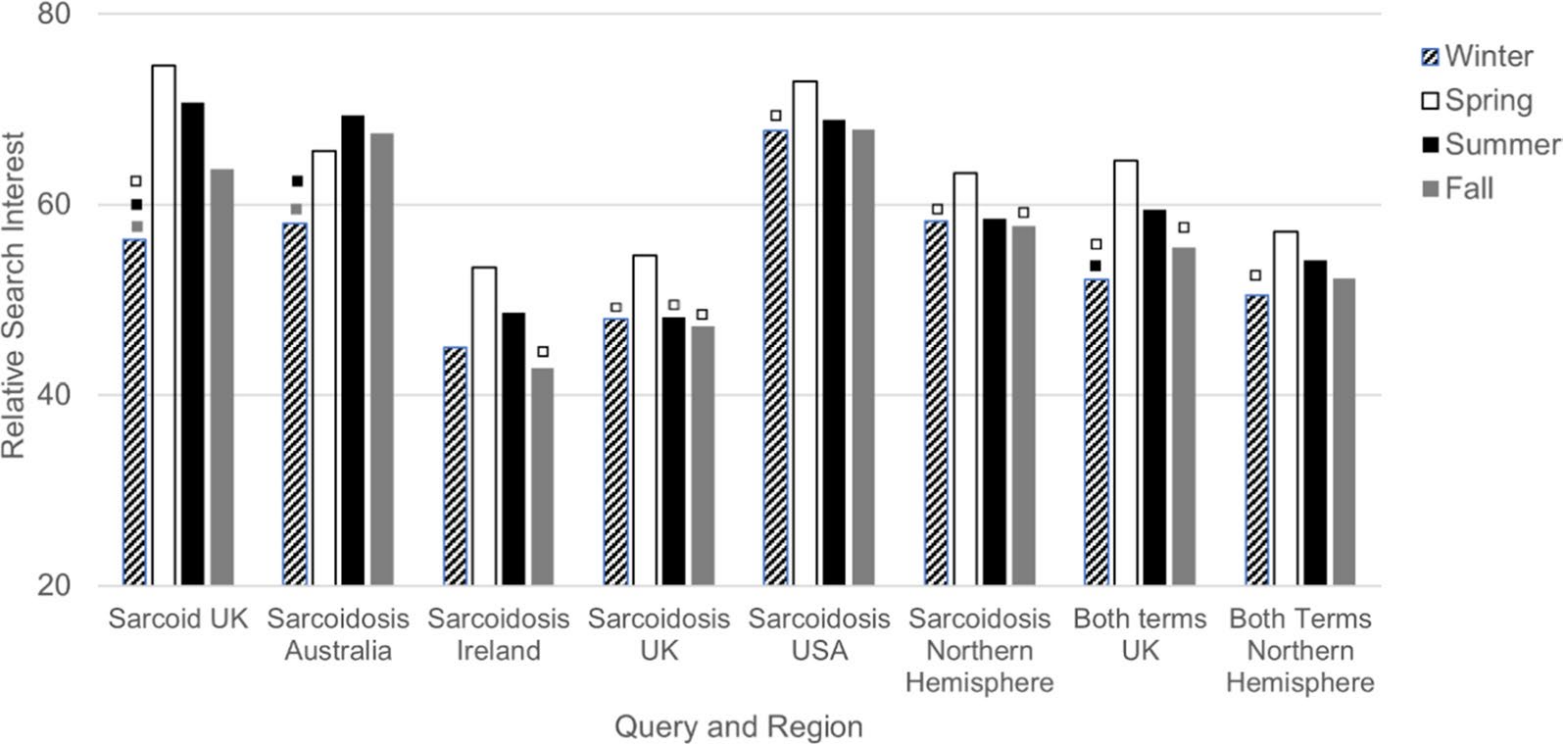
Google Trends search interest for regions with significant seasonal variation. Included are all the datasets for which a *p*-value of less than 0.05 was obtained on ANOVA, indicating statistically significant overall seasonal variation. The symbol “□” above a given bar indicates *p* < 0.05 for the comparison between that season and the associated season represented by the colour of the symbol

**Fig. 2:**
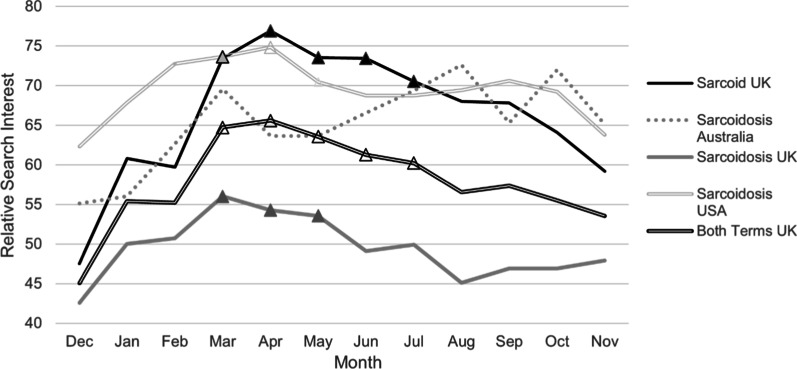
Google Trends search interest for regions with significant monthly variation. Included are all the datasets for which a *p*-value of less than 0.05 was obtained on ANOVA, indicating statistically significant overall monthly variation. Triangular markers indicate a *p*-value of < 0.05 for that month when compared to the associated December. Note that the marker for March, intersecting the “Sarcoid UK” and “Sarcoidosis USA” lines is slightly different because it represents both values showing statistical significance

Figures 1 and 2 show the results of the ANOVA and multiple comparisons that were statistically significant for the seasonal and monthly data, respectively. Relative search volume for “sarcoidosis” showed significant seasonal variation in Ireland (p = 0.02), the UK (p = 0.0002), Australia (p = 0.002), the USA (p = 0.03), and the Northern Hemisphere (p = 0.001). This was highest in March–May in the northern countries and lowest in November-February for all countries. The combined dataset revealed significantly higher relative search volume in spring compared to winter in the Northern Hemisphere (p = 0.004). Seasonal data from the UK showed lowest volume in the winter and highest in the summer in all three search term datasets.

Of note, December–February, summer months, were significantly less popular than both winter (June–August, p = 0.001) and spring (September–November, p = 0.01) in Australia. None of the results from the other southern countries analyzed were statistically significant, but there was a trend toward lower search volume in December, and, less markedly, July–August. Overall, September and October, spring months, were relatively popular in the southern countries.

With respect to monthly data, there was a statistically significant difference between months for the term “sarcoidosis” in Australia, the UK, and the USA. “Sarcoidosis” was more popular in March and April compared to the month of December in the USA and the UK. Furthermore, data for all three datasets from the UK strongly indicated overall variation (p = 0.0001–0.001), with relative search volume lowest in December and significantly different compared to the months of March to May. In addition, volume in December was significantly less than that of the months of June and July for “sarcoid” and both search terms combined.

## Discussion

The results of this analysis indicate that searches for sarcoidosis are higher in the spring months, and significantly less so in the winter, particularly in the Northern Hemisphere. This suggests that the condition may be more commonly diagnosed in the spring. Overall, this seems to support findings of higher incidence in winter-spring, as observed in previous studies. Both the Minnesota and the Taiwanese studies showed lowest incidence in autumn, which was statistically significant [6, 7]. The Taiwanese study also found that almost two-thirds of individuals presented with sarcoidosis in winter or early spring [7].

Our study assumes that patients only search for sarcoidosis online after contact with the healthcare system and when they are most unfamiliar with the term, that is, when they are first diagnosed. In addition, there is often a delay between when patients develop symptoms and diagnosis; a delay would systematically skew results. If sarcoidosis truly is most common in the winter-spring and least common in autumn, a few weeks’ delay would shift search patterns forward in time, making winter appear to be less popular, reflecting lower incidence in autumn, followed by the spring peak, corresponding to incidence in winter-early spring. Once they have been diagnosed, we expect that patients would preferably consult other sources, such as their physician, or resources provided to them by their physician, when their disease relapses. There are cases of seasonal variation in hypercalcemia in sarcoidosis patients, so it is plausible that this could also impact Google search trends in a similar fashion [14].

Another important assumption was that the countries included follow a traditional four-season structure. We would expect opposing results when comparing southern hemisphere countries to those in the northern hemisphere; that is, that June–August would have the least search term popularity and September–November, the most. The results of the comparison of means in the southern countries showed mixed trends, none of which met statistical significance. December was less popular overall, but a smaller peak in interest was observed in September and October. The December phenomenon suggests that the variation may be due, in part, to a non-weather-related factor, but the latter peak is more consistent with the pattern observed in the Northern Hemispheric countries and true seasonal variation.

The main limitation of our study was the nature of the data obtained from Google Trends. In the interest of maintaining privacy, Google Trends only provides relative search volume, and it is not possible to obtain absolute values. This makes it difficult to ascertain that the data is sufficient to make valid conclusions. Other countries with less access to internet or using search engines other than Google would be excluded, despite a relatively high prevalence of sarcoidosis. Lastly, the search terms used in this study were English words, which probably limited non-Anglophone countries from fulfilling the inclusion criteria.

As mentioned earlier, the results obtained from Google Trends varies day-by-day; this is because Google Trends only analyzes a sample of the total searches [9]. With billions of Google searches daily, it would take too long to analyze all of these searches [9]. This causes fluctuations that could become significant, especially in this case, where the term of interest is less popular. When search volume is too low, a value of zero is designated; zeroes were seen in our datasets, including those from Ireland, despite being noted as being the most popular country for both search terms in the worldwide data [9].

In Moccia et al.’s study on trends in Multiple Sclerosis, the country selection criteria were more stringent, with a minimum relative search volume of 70 and population of 20 million to avoid “noise” from areas of less search interest [12]. This was not feasible for our study since the prevalence of sarcoidosis is lower than that of Multiple Sclerosis [15, 16]; utilization of this criteria would have resulted in exclusion of more than half of the countries analyzed. However, higher prevalence may also lead to more searches by individuals other than patients, reducing the ability to correlate search volume with disease patterns, the major confounder in the Multiple Sclerosis study [12].

The strongest data to support the overall results was obtained from the United Kingdom, which showed very significant data in every data set. This probably swayed the overall statistics for the Northern Hemisphere countries towards significance. The incidence of sarcoidosis is about to 11.5/100,000 in Sweden [17], but only estimated at 7 per 100,000 in Great Britain [18]. Yet, Sweden did not meet the relative volume requirements for this analysis, so the data does not always represent burden of disease. One article looking at the use of Google Trends in epidemiology concluded that data “seems to be more influenced by the media clamor than by true epidemiological burden” [19]. The study looked at several medical conditions, including Ebola: it showed no concordance with the geographic or temporal patterns of this disease [19]. Most notably, there were two significant peaks in search interest for “Ebola” in Northern Italy in 2014 despite not a single case there [19]. Though this is an extreme example, it does not negate the need to be cautious with Internet-based data.

## Conclusions

Our study adds evidence to the hypothesis that the incidence of sarcoidosis exhibits seasonal variation and is more commonly diagnosed in the winter & spring. It is expected that future work will be needed in determining how to best incorporate “infodemiology” and the wealth of Internet-based data into future medical research. Hopefully, the results of this study lead to more investigation into the etiology behind this seasonal phenomenon and better understanding of the pathogenesis of sarcoidosis.

## Supplementary Information

**Additional file 1.** Raw search results for term "Sarcoidosis" by month and country.

**Additional file 2.** Raw search results for term "Sarcoid" by month and country.

## Data Availability

The dataset(s) supporting the conclusions of this article is(are) included within the article (and its additional file(s)).

## Abbreviations

UK: United Kingdom
USA: United States of America
